# Epigallocatechin‐3‐Gallate Ameliorates Diabetic Kidney Disease by Inhibiting the TXNIP/NLRP3/IL‐1β Signaling Pathway

**DOI:** 10.1002/fsn3.4617

**Published:** 2024-11-26

**Authors:** Yinghui Wang, Qimeng Wang, Mingming Wang, Xueling Wang, Qingzhen Liu, Shasha Lv, Huibin Nie, Gang Liu

**Affiliations:** ^1^ Department of Nephrology, Multidisciplinary Innovation Center for Nephrology The Second Hospital of Shandong University Jinan Shandong China; ^2^ Nephrology Research Institute of Shandong University Jinan Shandong China; ^3^ Department of Nephrology, Chengdu First People's Hospital Integrated TCM and Western Medicine Hospital Affiliated to Chengdu University of TCM Chengdu Sichuan China; ^4^ Key Laboratory of Reproductive Endocrinology of Ministry of Education Shandong University Jinan Shandong China

**Keywords:** diabetic kidney disease, EGCG, green tea, inflammation, NLRP3, TXNIP

## Abstract

Recent research indicates that the activation of the NLRP3 inflammasome is crucial in the development of diabetic kidney disease (DKD). Epigallocatechin‐3‐gallate (EGCG), the predominant catechin in green tea, has been noted for its anti‐inflammatory properties in DKD. However, the specific mechanisms are not yet fully understood. In this study, our objective was to explore the effects of EGCG on podocytes and in diabetic kidney disease (DKD) mice and investigate how EGCG modulates the TXNIP/NLRP3/IL‐1β signaling pathway in DKD, both in podocytes and animal models. In vitro, we co‐cultured podocytes with EGCG and detected the viability, apoptosis, inflammation and the TXNIP/NLRP3/IL‐1β signaling pathway. In vivo, DKD mice were given EGCG via oral gavage, followed by evaluations of renal function, inflammation, and the aforementioned signaling pathway. Our findings revealed that oxidative stress, inflammatory cytokines, and the TXNIP/NLRP3/IL‐1β pathway were upregulated in podocytes exposed to high glucose (HG) and in the kidneys of DKD mice. However, EGCG treatment reduced the expression of the NLRP3 inflammasome and its associated proteins, including TXNIP, ASC, caspase‐1, and IL‐1β, as well as the levels of ROS and inflammatory factors such as TNF‐α, IL‐6, and IL‐18. Furthermore, in vivo, EGCG improved kidney function, reduced albuminuria and body weight, and alleviated renal pathological damage. In summary, our study suggests that EGCG mitigates inflammation in podocytes and DKD through the TXNIP/NLRP3/IL‐1β signaling pathway, indicating potential benefits of EGCG or green tea in managing DKD.

## Graphical abstract

NLRP3 inflammasome‐mediated inflammation plays an important role in the pathogenesis and progression of DKD. EGCG is the most abundant catechin in green tea and has anti‐inflammatory effects in DKD. Our research demonstrated that EGCG protects against DKD through suppression of inflammation via the TXNIP/NLRP3/IL‐1β signaling pathway. Our findings suggest that EGCG or green tea can be beneficial in the regulation of DKD.

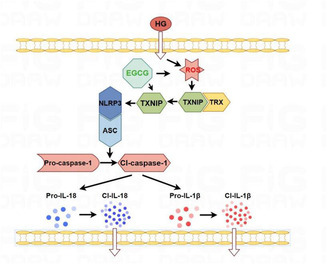

**Abbreviations**
AGEs: advanced glycation end products
ASC: apoptosis‐associated speck‐like protein containing a caspase recruitment domain
CAT: catalase
DAPA: dapagliflozin
DHE: dihydroethidium
DKD: diabetic kidney disease
DM: diabetes mellitus
EGCG: epigallocatechin‐3‐gallate
ESRD: end‐stage renal disease
FBG: fasting blood glucose
FPW: foot process width
GSH: glutathione
HG: high glucose
IF: immunofluorescence
IL: interleukin
Man: mannitol
MDA: malondialdehyde
MMP: mitochondrial membrane potential
NLRP3: nucleotide‐binding domain‐like receptor 3
PAS: periodic acid Schiff
RAAS: renin–angiotensin–aldosterone system
ROS: reactive oxygen species
SOD: superoxide dismutase
TEM: transmission electron microscopy
TXNIP: thioredoxin‐interacting protein
WT‐1: wilms tumor‐1

## 1 Background

Diabetic kidney disease (DKD) is a chronic microvascular complication linked to diabetes mellitus (DM) and is the primary cause of end‐stage renal disease (ESRD) (Naaman and Bakris 2023). It is marked by declining renal function, worsening albuminuria, and hypertension. Despite current treatments, such as effective blood pressure and glycemic control, sodium‐glucose cotransporter 2 inhibitors, and renin–angiotensin–aldosterone system (RAAS) blockade, approximately 40% of diabetic patients still progress to DKD (Vartak, Godson, and Brennan 2021; Stanton 2014). Therefore, identifying new and effective therapies to delay DKD progression is of critical importance.

In recent years, increasing evidence has shown that sterile inflammation, mediated by the NLRP3 inflammasome, significantly contributes to the development and progression of DKD (Tang and Yiu 2020; Qiu and Tang 2016). Studies have shown that kidney inflammation and the substantial release of chemokines and inflammatory factors were strongly correlated with the development of hyperglycemia and albuminuria in DKD (Chen et al. 2019). NLRP3 inflammasome recruits ASC and pro‐caspase1. Upon activation, caspase1 cleaves the pro‐inflammatory cytokines pro‐IL‐1β and pro‐IL‐18 into their mature, biologically active forms, which drive the inflammatory response (Kim et al. 2019; Yaribeygi et al. 2019). Shahzad et al. demonstrated that the absence of NLRP3 or caspase1 ameliorated albuminuria and reduced the fractional mesangial area in DKD mice (Shahzad et al. 2015). Similarly, Zhang et al. found that MCC950, an NLRP3 inflammasome inhibitor, preserved renal function, reduced podocyte injury, and mitigated renal fibrosis in diabetic mice (Zhang et al. 2019). Although the NLRP3 signaling pathway is critical to the sterile inflammation of DKD, the precise mechanism by which the NLRP3 inflammatory complex leads to renal injury in DKD remains unclear.

Thioredoxin‐interacting protein (TXNIP), a mediator of oxidative stress, has been shown to interact with and activate the NLRP3 inflammasome (Schroder, Zhou, and Tschopp 2010; Zhang et al. 2015). Studies have reported significant upregulation of TXNIP and NLRP3 expression in DKD mice (Wang et al. 2023a). Haidy et al. found that calycosin could alleviate the progression of DKD by mediating both NLRP3 and TXNIP (Yosri et al. 2022). Han et al. further revealed that the mitochondrial reactive oxygen species (ROS)‐TXNIP/NLRP3/IL‐1β axis is activated in tubular HK‐2 cells, with the TXNIP/NLRP3/IL‐1β axis playing an essential role in tubular oxidative injury (Han et al. 2018).

Epigallocatechin‐3‐gallate (EGCG), the prominent catechin in green tea, is recognized for its high antioxidant and anti‐inflammatory potential (Higdon and Frei 2003; Rashidinejad et al. 2021; Butt and Sultan 2009). Studies revealed that oral administration of EGCG or green tea can prevent insulin resistance against diabetes as well as associated complications, including DKD, in diabetic animal models (Shahwan et al. 2022; Babu, Liu, and Gilbert 2013; Sun et al. 2017). Di et al. indicated that EGCG could alleviate inflammation and oxidative stress via the SIRT1/NLRP3 (Di et al. 2022). Mohan et al. reported that EGCG treatment inhibited the expression of TGF‐β and enhanced renal functions in DKD (Mohan et al. 2017). Furthermore, evidence suggests that EGCG may interact with the NLRP3 inflammasome (Wang et al. 2021, 2020). Thus, we hypothesize that EGCG may exhibit anti‐inflammatory activities through the TXNIP/NLRP3/IL‐1β axis both in podocytes and DKD mice.

In this research, we first evaluated the impact of EGCG in cell and animal models. We then tested the hypothesis that EGCG exerts anti‐inflammatory activities via the TXNIP/NLRP3/IL‐1β axis in mouse models and podocytes.

## 2 Materials and Methods

### 2.1 Cell Cultures and Treatments

Human podocytes, generously provided by Prof. Fan Yi (Shandong University), were initially cultured at 33°C for proliferation and then shifted to 37°C for a 2‐week differentiation period. Subsequently, the podocytes were maintained in RPMI1640 medium containing 1% penicillin–streptomycin and 10% FBS (HyClone, USA) at 37°C and 5% CO_2_/95% air. Upon reaching 80% confluence, they were randomly assigned to 4 groups: (1) normal group (5.5 mM D‐glucose for 48 h, CON group); (2) osmotic control group (24.5 mM D‐mannitol and 5.5 mM D‐glucose for 48 h, Man group); (3) high glucose group (30 mM D‐glucose for 48 h, HG group); (4) high glucose + EGCG group (≥ 98% purity, Solarbio, China) (firstly cultured with 30 μM EGCG for 24 h, followed by 30 mM D‐glucose for 48 h).

### 2.2 Measurement of Podocyte Viability

Podocyte viability was examined using a cell counting kit‐8 (Dojindo, Japan). Briefly, podocytes were grown in 96‐well plates, and after the respective treatments, CCK‐8 solution (10 μL) and RPMI1640 medium (90 μL) were added. The plates underwent incubation for 2 h. The absorbance was recorded using a microplate reader at 450 nm.

### 2.3 Measurement of Podocyte Apoptosis

The apoptosis rate of podocytes was determined using an AnnexinV‐FITC kit (BD Bioscience, USA). Briefly, following various treatments, the podocyte sediments were resuspended in 100 μL of AnnexinV/PI binding buffer. Then, Annexin V‐FITC (5 μL) and PI (5 μL) were added, followed by a 30‐min incubation at ambient temperature in darkness. Flow cytometric analysis was conducted to analyze the samples within 1 h.

### 2.4 Measurement of Oxidative Stress in Podocytes

Podocyte intracellular ROS levels were measured by dihydroethidium (DHE, Beyotime, China). Podocytes were exposed to 10 μM DHE at 37°C for 30 min for DHE staining and subsequently examined using a Zeiss‐LSM800‐laser‐scanning‐confocal‐microscope (Zen, Germany).

Mitochondrial membrane potential (MMP) was determined by Mito Tracker Red CMXRos (Biotime, China). Briefly, after washing with PBS, podocytes underwent incubation with Mito‐Tracker‐Red‐CMXRos solution for 30 min at 37°C. Images were obtained using the aforementioned microscope (Zen, Germany).

### 2.5 Animal Treatments

All the in vivo protocols received approval from the Animal Ethics Committee of the Second Hospital of Shandong University (IACUC approval no: KYLL‐2023‐411). All methods complied with the relevant guidelines and regulations set forth by the Animal Ethics Committee and followed ARRIVE guidelines. Briefly, 8‐week‐old C57BLKS/J male db/db mice (type 2 diabetes mode; weight: 31.46 ± 4.51) and normal control male mice (db/m mice; weight: 19.35 ± 3.82) were all specific pathogen‐free (SPF), purchased from XingKangshengwu (Jinan, China). Db/db mice were classified as having DKD if fasting blood glucose (FBG) levels exceeded 15 mmol/L and urinary protein tests were positive. Then, the 10‐week‐old DKD mice were assigned to 3 groups for treatment (*n* = 4, each): (1) DKD group (oral administration of 0.9% saline via gavage); (2) DKD + EGCG group (oral administration of 100 mg/kg/day EGCG via gavage); (3) DKD + dapagliflozin (DAPA, AstraZeneca, UK) group (oral administration of 1 mg/kg/day DAPA via gavage). Db/m mice served as normal controls (oral administration of 0.9% saline via gavage) (CON group, *n* = 4). After completing the study, mice underwent anesthetization with pentobarbital (50 mg/kg) via intraperitoneal injection. One kidney was promptly frozen in liquid nitrogen and kept at −80°C for molecular studies. The other kidney was either fixed in paraformaldehyde (4%) for histopathological examination or sliced into 1 mm^3^ sections and preserved in chilled glutaraldehyde (5%) for electron microscopy. After the collection of tissues and samples, mice were euthanized through cervical dislocation at week 18. Fasting blood glucose (FBG) and urine albumin of mice were measured as previously described (Wang et al. 2023b).

### 2.6 Measurement of Inflammatory Cytokine

After collected from ophthalmic vein and centrifuged at 10,000 rpm for 10 min, blood samples were stored at −80°C. The concentrations of inflammatory factors in cell supernatants and mouse serum samples were measured using ELISA kits (Thermo Fisher Scientific, USA) following the manufacturer's instructions.

### 2.7 RT‐qPCR Assay

Total RNA from kidney tissues and podocytes was extracted by Trizol reagent (Invitrogen, USA). cDNA was prepared by a reverse transcription kit (TaKaRa, China), then cDNA was quantified by SYBR Green PCR Master Mix (TaKaRa, China). A PCR instrument (ABI, USA) was used to perform the real‐time reaction. Relative levels of mRNA were calculated by the $2^{-\Delta \Delta C_{T}}$ method. The specific primer sequences used are described in Table 1.

**TABLE 1:** Primer sequences for RT‐qPCR.

Gene	Forward primer (5′–3′)	Reverse primer (5′–3′)
*NLRP3* (Human)	TGGATGGGTTTGCTGGGAT	CTGCGTGTAGCGACTGTTGAG
*NLRP3* (Mouse)	ATTACCCGCCCGAGAAAGG	TCGCAGCAAAGATCCACACAG
*Caspase‐1* (Human)	TTTCCGCAAGGTTCGATTTTCA	GGCATCTGCGCTCTACCATC
*Caspase‐1* (Mouse)	ACAAGGCACGGGACCTATG	TCCCAGTCAGTCCTGGAAATG
*IL‐1β* (Human)	ACCTTCCAGGATGAGGACATGA	AACGTCACACACCAGCAGGTTA
*IL‐1β* (Mouse)	GAAATGCCACCTTTTGACAGTG	TGGATGCTCTCATCAGGACAG
*ASC* (Human)	GACTGCACTTTGTGGACCAG	AGCAGATCCTTGCAGGTCAT
*ASC* (Mouse)	GCAATGTGCTGACTGAAGGA	TGTTCCAGGTCTGTCACCAA
*TXNIP* (Human)	TGTGTGAAGTTACTCGTGTCAAA	GCAGGTACTCCGAAGTCTGT
*TXNIP* (Mouse)	TCAATACCCCTGACCTAATGGC	TTCTGTCAATTCGAGCAGAGAC
*IL‐6* (Mouse)	CTGCAAGAGACTTCCATCCAG	AGTGGTATAGACAGGTCTGTTG
*TNF‐α* (Mouse)	CTGAACTTCGGGGTGATCGG	GGCTTGTCACTCGAATTTTGA
*IL‐18* (Mouse)	GTGAACCCCAGACCAGACTG	CCTGGAACACGTTTCTGAAAGA
*GAPDH* (Human)	GGAGCGAGATCCCTCCAAAAT	GGCTGTTGTCATACTTCTCATGG
*GAPDH* (Mouse)	TGGCCTTCCGTGTTCCTAC	GAGTTGCTGTTGAAGTCGCA

### 2.8 Immunoblotting

Total protein in kidney tissues and podocytes was extracted by RIPA lysis buffer, and the protein content was measured using a protein assay kit (Beyotime, China). After being heated with 5 × SDS‐PAGE loading buffer, 40 μg of protein (per sample) were electrophoresed to 12% SDS‐PAGE gels. The separated proteins were then transferred onto PVDF membranes (Millipore, USA). Then, the membranes were incubated overnight with primary antibodies, including anti‐NLRP3 (1:1000, CST), anti‐TXNIP (1:2000, Abcam), anti‐Pro‐caspase‐1 (1:1000, Sigma, USA), anti‐Cl‐caspase‐1 P20 (1:500, Sigma, USA), anti‐Pro‐IL‐1β (1:1000, Abcam, USA), anti‐Cl‐IL‐1β P17 (1:200, Abcam, USA), anti‐ASC (1:1000, Abcam, USA), anti‐GAPDH (1:3000; Zsbio, China), and anti‐β‐Actin (1:3000, Zsbio, China) at 4°C. After that, the membranes were incubated for 1 h with a secondary antibody (1:3000, goat anti‐rabbit/mouse, Zsbio, China). A WB instrument (Tanon, China) was used to visualize the bands. The data were analyzed using ImageJ software.

### 2.9 Histopathology of Kidney

Kidney tissue samples were fixed in paraformaldehyde (4%), embedded in paraffin, and then sectioned (thickness = 4 μm) for histopathological analysis. Periodic acid‐Schiff (PAS) and Masson staining were conducted to evaluate renal pathological changes, following the kit's protocols (Solarbio, China).

### 2.10 Measurement of Oxidative Stress in Kidney

The kidney tissues were homogenized in PBS, followed by centrifugation at 10,000 *g* for 10 min at 4°C. The supernatants were harvested and analyzed for oxidative stress markers, including glutathione (GSH) levels, malondialdehyde (MDA) content, and the activities of catalase (CAT) and superoxide dismutase (SOD), using commercial kits (Beyotime, China).

### 2.11 Transmission Electron Microscopy (TEM)

The kidney tissue sample preparation and TEM analysis were performed by the Shandong Normal University electron microscopic core lab. The ultrastructural changes in kidneys were measured by Image J software. The glomerular basement membrane (GBM) thickness, foot process number (FPN), and foot process width (FPW) were computed as described (Deegens et al. 2008).

### 2.12 Immunofluorescence (IF)

Cells and kidney samples were firstly exposed to phalloidin (1:100; Sharebio, China) and primary antibodies, including anti‐synaptopodin (1:300; Sigma, USA), anti‐nephrin (1:500; Abcam, USA), anti‐WT‐1 (1:50; Abcam), anti‐NLRP3 (1:200; Abcam), anti‐caspase‐1 (1:500; Abcam), and anti‐ASC (1:200; Abcam) at 4°C overnight. The kidney tissues were then exposed to FITC/rhodamine‐conjugated secondary antibody (1:100, Zsbio) for 1 h. The cell nuclei were stained with DAPI (1:1000, Solarbio) for 5 min. Images were captured using the aforementioned microscope.

### 2.13 Statistical Analysis

Statistical tests were conducted using GraphPad Prism8. Data are presented as means±SD. Two group comparisons were performed by t‐tests, while comparisons among multiple groups were conducted by one‐way ANOVA and Tukey's post hoc test. *p* < 0.05 was deemed statistically significant.

## 3 Results

### 3.1 EGCG Increased Podocyte Viability and Protected Podocytes From Apoptosis

**FIGURE 1:**
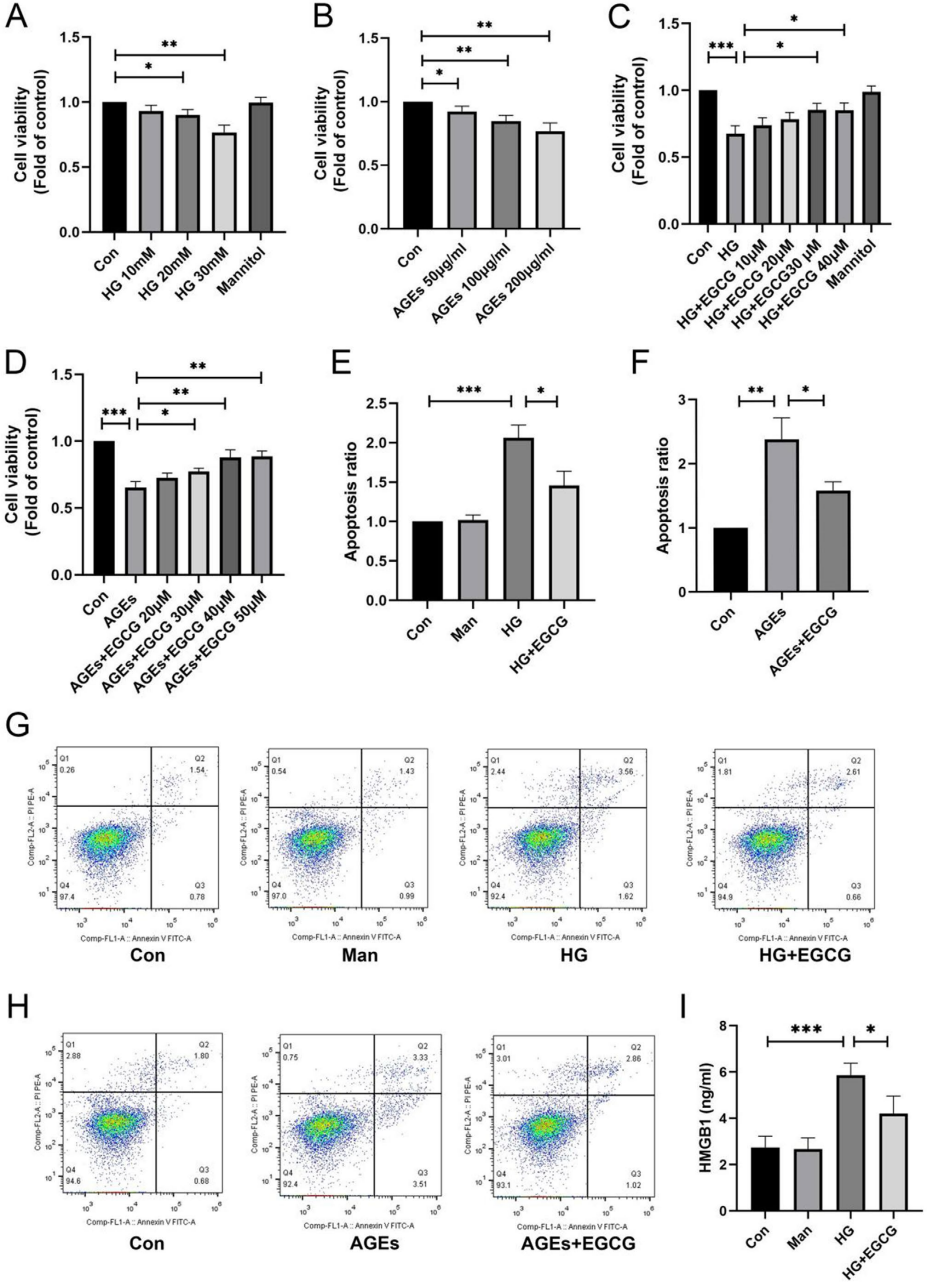
Protective impact of MSCs‐Exo on podocytes. (A, B) Viability of podocytes exposed to varying concentrations of AGEs and HG for 48 h (*n* = 3). (C, D) Podocyte viability following treatment with escalating concentrations of EGCG under HG (30 mM) and AGEs (200 μg/mL) for 48 h (*n* = 3). (E, G) Flow cytometric analysis summarizing podocyte apoptosis in response to 30 mM HG, with or without 30 μM EGCG, over 48 h (*n* = 3). (F, H) Flow cytometric analysis summarizing podocyte apoptosis in response to 200 μg/mL AGEs, with or without 40 μM EGCG, over 48 h (*n* = 3). (I) HMGB1 expression levels in podocytes exposed to 30 mM HG with or without 30 μM EGCG for 48 h. Values are shown as means±SD. ***p < 0.001, **p < 0.01, *p < 0.05.

To determine the impact of EGCG on podocyte viability, we conducted a cell viability assay. Firstly, we evaluated podocyte viability under varying concentrations of HG and advanced glycation end products (AGEs), as illustrated in Figure 1A,B. Our findings revealed that podocyte viability reduced with elevating concentrations of HG and AGEs. Subsequently, we co‐cultured podocytes with different concentrations of EGCG in the presence of HG or AGEs. We observed that EGCG improved podocyte viability, particularly at concentrations of 200 μg/mL AGEs and 30 mM HG (Figure 1C,D). Moreover, as shown in Figure 1E–H, EGCG suppressed podocyte apoptosis caused by HG and AGEs as well as the pyroptosis marker HMGB1 (Figure 1I).

### 3.2 EGCG Decreased Oxidative Stress in Podocytes

**FIGURE 2:**
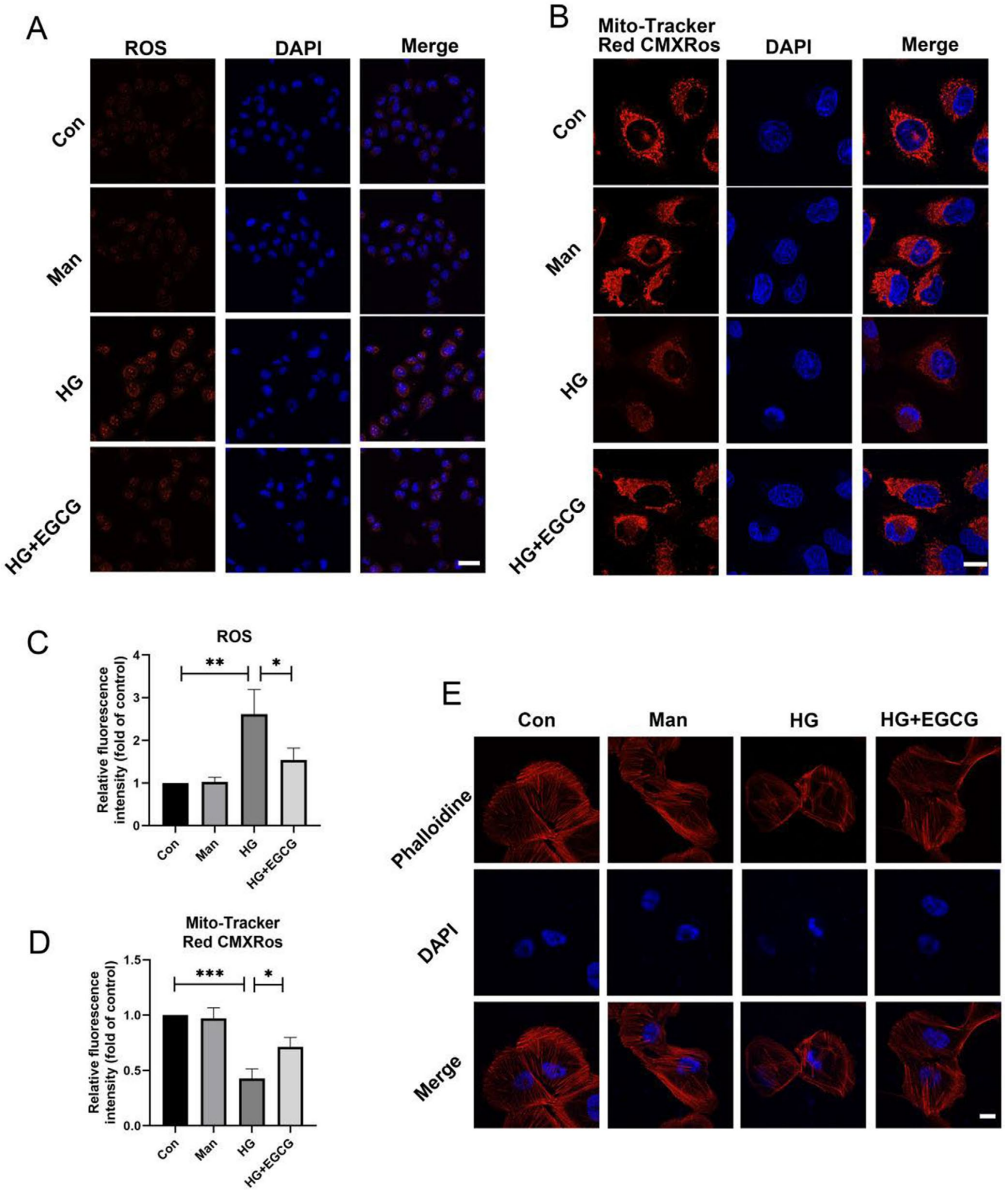
Oxidative stress and F‐Actin expression in podocytes. (A, C) Representative DHE staining and corresponding data illustrating ROS production in podocytes after various treatments for 48 h (EGCG: 30 μM; HG: 30 mM; *n* = 3, bar = 20 μm). (B, D) Representative MitoTracker Red CMXRos staining and corresponding data depicting mitochondrial membrane potential (MMP) in podocytes following various treatments for 48 h (EGCG: 30 μM; HG: 30 mM; *n* = 3, bar = 5 μm). (E) Representative confocal images of F‐actin in podocytes and nuclei stained with phalloidin (red) and DAPI (blue), respectively, after various treatments for 48 h (EGCG: 30 μM; HG: 30 mM; *n* = 3, bar = 5 μm). Values are shown as means ± SD, with significance levels indicated by ***p < 0.001, **p < 0.01, *p < 0.05.

DHE staining demonstrated that HG elevated ROS levels in podocytes compared to normal glucose, while EGCG reduced ROS levels under 30 mM HG conditions (Figure 2A–C). Mito‐Tracker Red CMXRos was used to specifically label bioactive mitochondria and assess MMP. We found that HG decreased the MMP of podocytes while EGCG increased the podocyte MMP (Figure 2B–D). Our results indicated that EGCG could suppress the oxidative stress in podocytes under HG. Additionally, immunofluorescence analysis indicated that HG induced changes in the cytoskeleton, whereas EGCG ameliorated cytoskeleton rearrangement under 30 mM HG conditions (Figure 2E).

### 3.3 EGCG Attenuated the Interactions Between TXNIP and NLRP3

**FIGURE 3:**
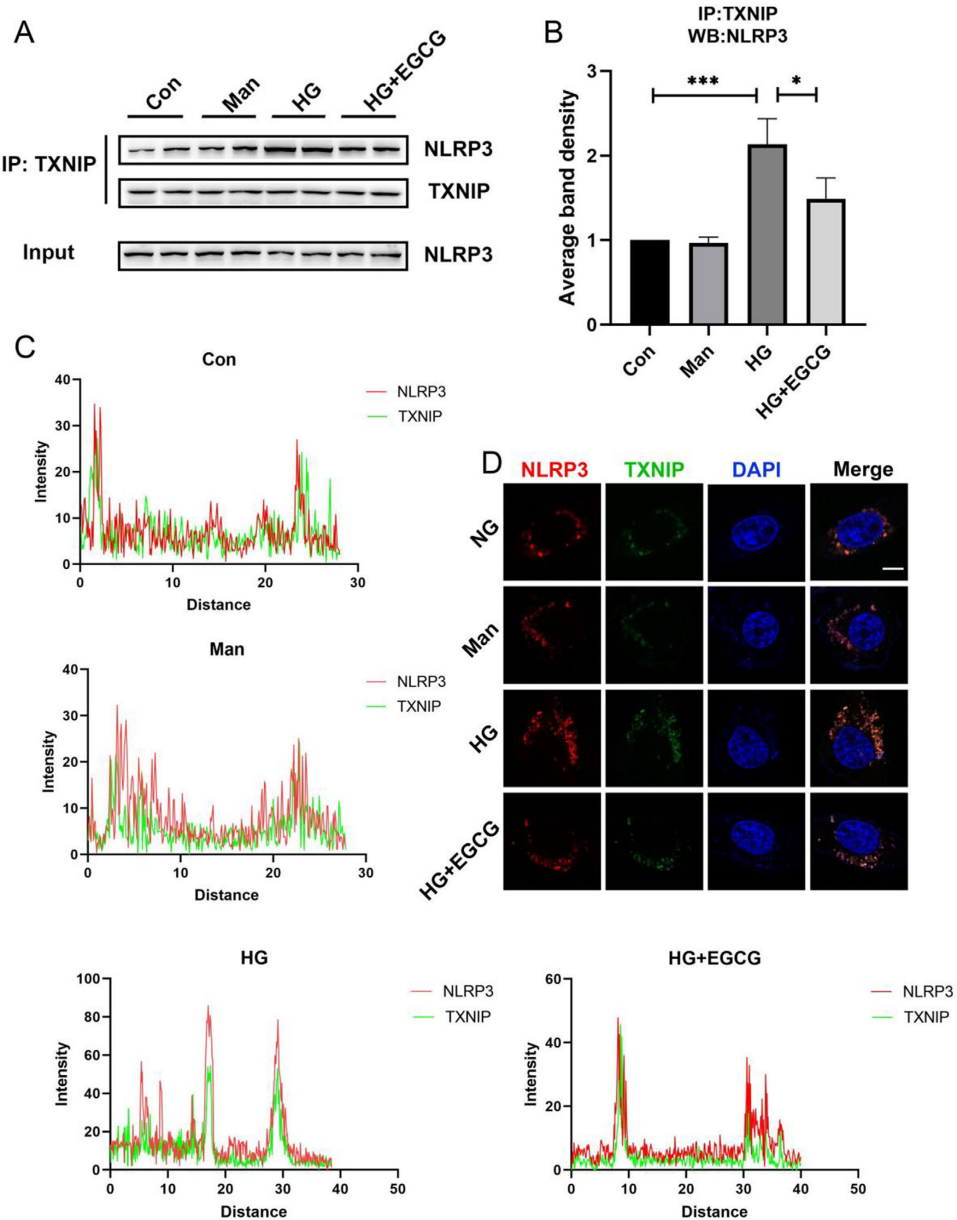
Relationship between TXNIP and NLRP3 expression. (A, B) Representative Co‐IP/immunoblot images and corresponding data illustrating the interactions between NLRP3 and TXNIP in podocytes exposed to HG (30 mM) and EGCG (*n* = 4). (C, D) Representative immunofluorescence staining and corresponding data depicting the co‐localization of TXNIP and NLRP3 in podocytes exposed to HG (30 mM) and EGCG (*n* = 3, bar = 5 μm). Values are shown as means ± SD. ***p < 0.001, *p < 0.05.

TXNIP interacts with NLRP3, promoting the initiation of the NLRP3 inflammatory complex. Research indicates that the NLRP3 inflammasome is activated in DKD. Thus, we investigated the increased interactions between TXNIP and NLRP3 in podocytes exposed to a HG environment. As shown in Figure 3A,B, co‐immunoprecipitation assays with NLRP3 and TXNIP antibodies revealed a significant increase in TXNIP‐NLRP3 interaction in HG‐treated podocytes. This enhanced binding was notably inhibited by EGCG treatment. Additionally, immunofluorescence staining demonstrated co‐localization of NLRP3 and TXNIP in podocytes under HG conditions (30 mM), which was mitigated by EGCG treatment (Figure 3C,D).

### 3.4 EGCG Suppresses HG‐Induced Inflammation in Podocytes via the TXNIP/NLRP3/IL‐1β Axis

**FIGURE 4:**
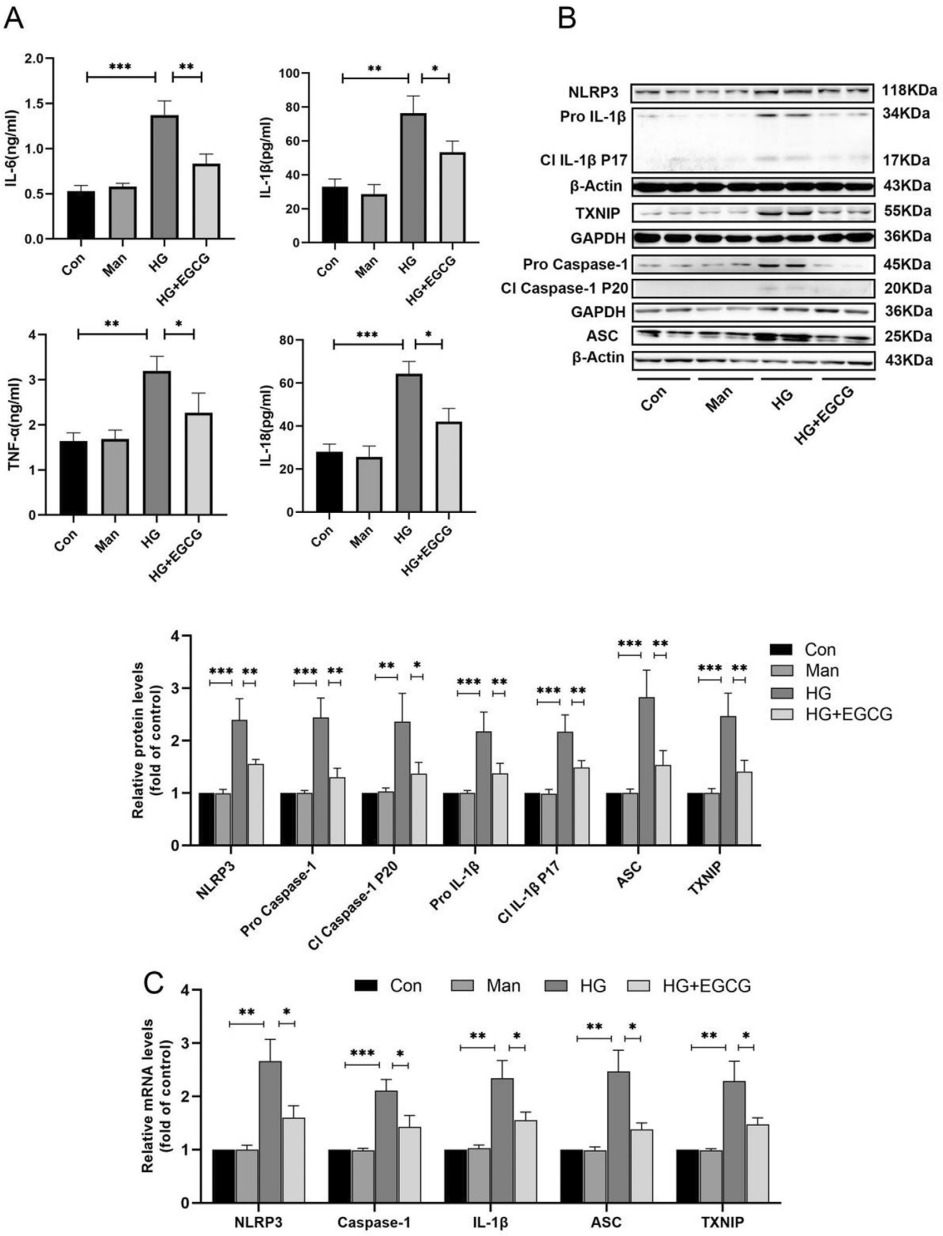
Impact of EGCG on the TXNIP/NLRP3/IL‐1β axis in podocytes. (A) Expression levels of inflammatory cytokines (TNF‐α, IL‐6, IL‐1β, IL‐18) in podocytes after various treatments for 48 h (EGCG: 30 μM; HG: 30 mM; *n* = 3). (B) Representative immunoblot images and corresponding data indicating the protein levels of NLRP3, TXNIP, caspase1, ASC, and IL‐1β in podocytes following various treatments for 48 h (EGCG: 30 μM; HG: 30 mM for 48 h; *n* = 4). (C) Relative mRNA levels of TXNIP, NLRP3, ASC, caspase1, and IL‐1β in podocytes after various treatments for 48 h (EGCG: 30 μM; HG: 30 mM for 48 h; *n* = 3). Values are shown as means ± SD, with significance levels indicated by ***p < 0.001, **p < 0.01, *p < 0.05.

Growing evidence indicates that inflammation is crucial in the occurrence of podocyte injury. To investigate whether EGCG impacts HG‐induced inflammation in podocytes, we measured the levels of TNF‐α, IL‐6, IL‐1β, IL‐18, and HMGB1 in cell supernatants through ELISA. As displayed in Figure 4A, pretreatment with EGCG markedly decreased the upregulation of TNF‐α, IL‐6, IL‐1β, IL‐18, and HMGB1 induced by HG treatment. We further examined the impact of EGCG on NLRP3 inflammasome activation and found that EGCG decreased both gene and protein expression of key signaling proteins in the TXNIP/NLRP3/IL‐1β axis, including TXNIP, ASC, NLRP3, caspase1, and IL‐1β (Figure 4B–D). These findings indicate that EGCG effectively suppresses HG‐induced inflammation in podocytes and suppresses NLRP3 inflammasome activation by modulating the TXNIP/NLRP3/IL‐1β axis.

### 3.5 EGCG Alleviates Oxidative Stress and Biochemical Indicators in the Kidneys

**FIGURE 5:**
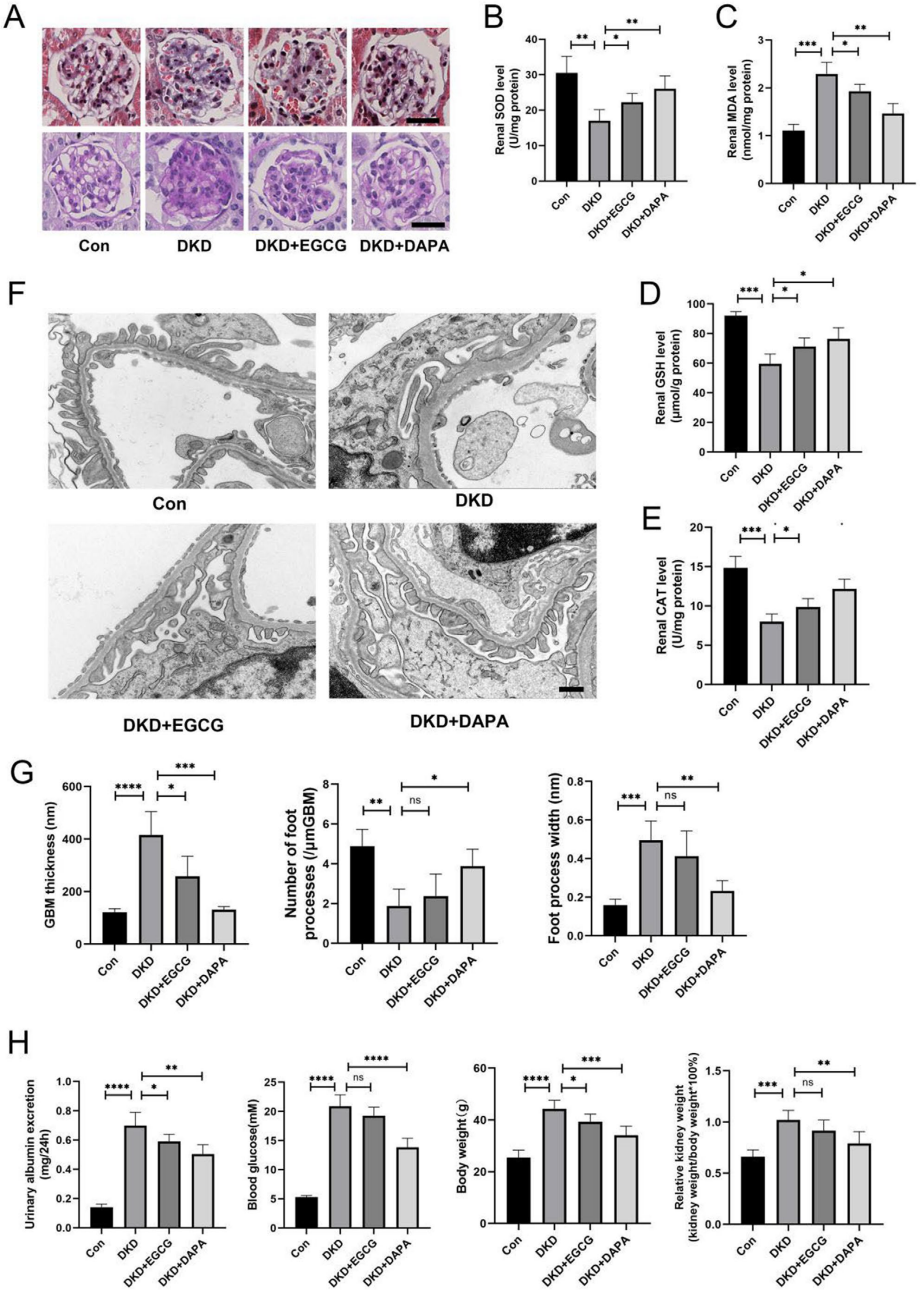
EGCG protects against kidney tissue damage in DKD mice. (A) Representative images of PAS and masson staining highlighting the glomerular structure alterations across diverse groups (*n* = 4, bar = 20 μm). (B) SOD activity in kidney homogenates among various groups (*n* = 4). (C) MDA content in kidney homogenates among various groups (*n* = 4). (D) GSH levels in kidney homogenates among various groups (*n* = 4). (E) CAT activity in kidney homogenates among various groups (*n* = 4). (F, G) Representative TEM images of the glomerular filtration barrier and corresponding data for GBM thickness, FPN, and FPW across diverse groups (*n* = 4, bar = 0.5 μm). (H) Urinary albumin excretion, blood glucose, body weight, and relative kidney weight across diverse groups (*n* = 4). Values are shown as means ± SD, with significance levels indicated by ^****^ *p* < 0.0001, ***p < 0.001, **p < 0.01, *p < 0.05, ns: Not significant.

Research has highlighted the critical role of oxidative stress in kidney injury associated with DKD. We have previously demonstrated that EGCG can reduce oxidative stress in podocytes under HG conditions. To further explore the potential antioxidant effects of EGCG on the kidneys in vivo, we examined its impact on DKD mice. As expected, DKD mice exhibited significantly elevated levels of MDA, reduced activities of SOD and CAT, and declined levels of GSH compared to the normal control group. EGCG treatment, particularly in combination with DAPA, significantly elevated GSH, CAT, and SOD levels while declining MDA levels in the kidneys of DKD mice. This suggests that EGCG effectively mitigates oxidative stress in DKD (Figure 5B–E).

As shown in Figure 5H, DKD mice had significantly higher urinary albumin excretion, blood glucose levels, body weight, and relative kidney weight compared to normal control mice. EGCG treatment reduced urinary albumin excretion and body weight in DKD mice compared to untreated DKD mice, although no obvious differences were noted in blood glucose levels or relative kidney weight between the untreated and EGCG‐treated DKD mice.

### 3.6 EGCG‐Alleviated Renal Injury in DKD Mice

To examine the histopathological changes in the kidneys, we performed PAS and Masson staining. Figure 5A illustrates that PAS staining revealed significant glomerular mesangial matrix accumulation in DKD mice compared to normal controls. This accumulation was alleviated in DKD mice treated with EGCG and DAPA. Masson staining was used to assess renal interstitial fibrosis, as shown in Figure 5A. Masson staining demonstrated that DKD mice treated with EGCG and DAPA exhibited a marked reduction in fibrosis compared to the untreated DKD group. Additionally, TEM showed that EGCG treatment reduced the thickening of GBM. However, no obvious differences were noted in the density of podocyte foot processes between DKD mice and EGCG‐treated DKD mice (Figure 5F,G).

### 3.7 EGCG Suppresses Inflammation in DKD Mice via the TXNIP/NLRP3/IL‐1β Axis

**FIGURE 6:**
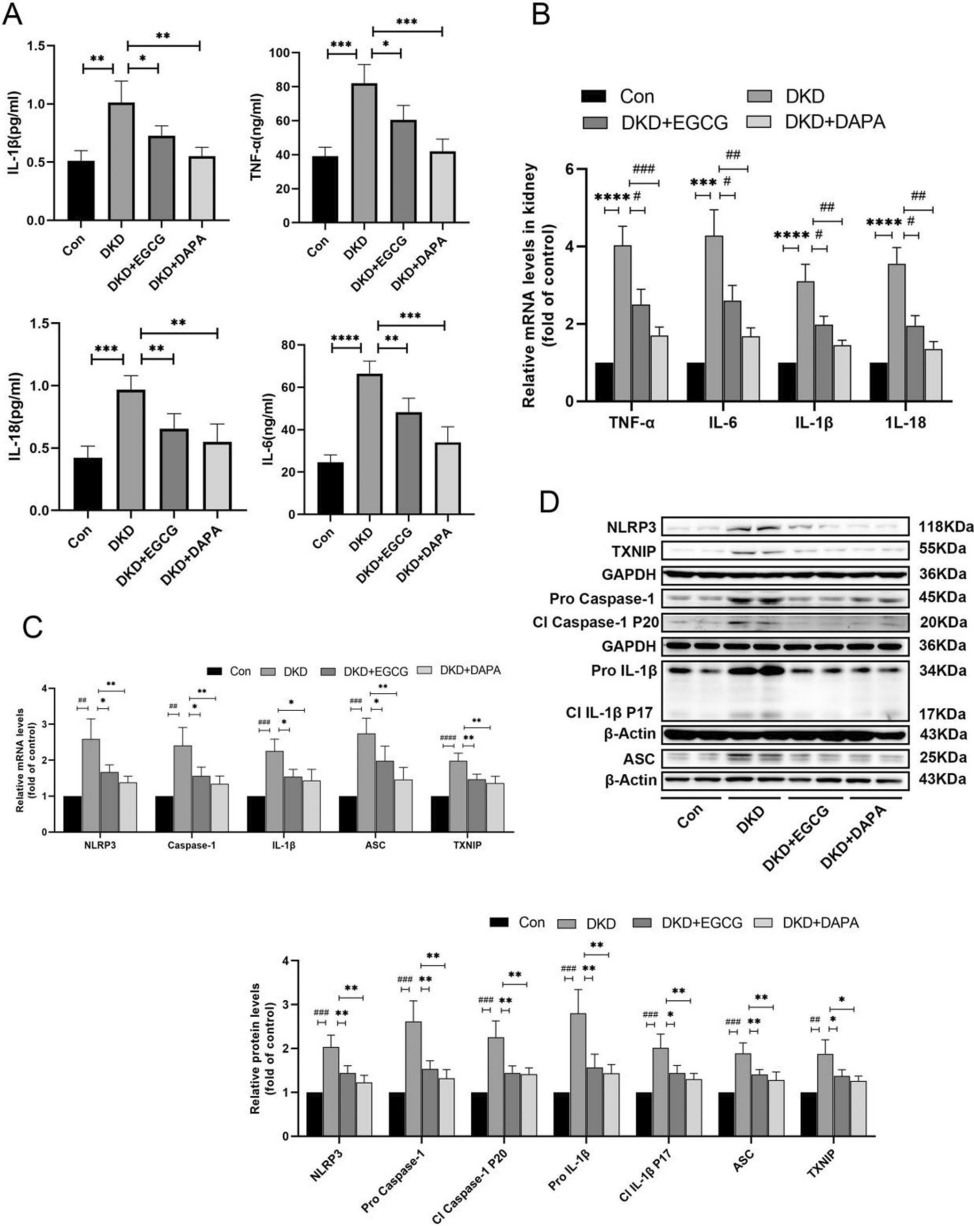
Effects of EGCG on the TXNIP/NLRP3/IL‐1β axis in DKD kidneys. (A) Expression levels of inflammatory cytokines (TNF‐α, IL‐6, IL‐1β, IL‐18) in serum across diverse groups (*n* = 4). (B) mRNA levels of inflammatory cytokines (TNF‐α, IL‐6, IL‐1β, IL‐18) in renal tissues across various groups (*n* = 4). (C) Relative mRNA expression of NLRP3, TXNIP, caspase1, ASC, and IL‐1β among various groups (*n* = 4). (D) Representative immunoblot images and corresponding data demonstrating the protein levels of NLRP3, TXNIP, caspase1, ASC, and IL‐1β among various groups (*n* = 4). Values are shown as means±SD. ^****^ *p* < 0.0001, ***p < 0.001, **p < 0.01, *p < 0.05, ^####^ *p* < 0.0001, ^###^ *p* < 0.001, ^##^ *p* < 0.01, ^#^ *p* < 0.05.

**FIGURE 7:**
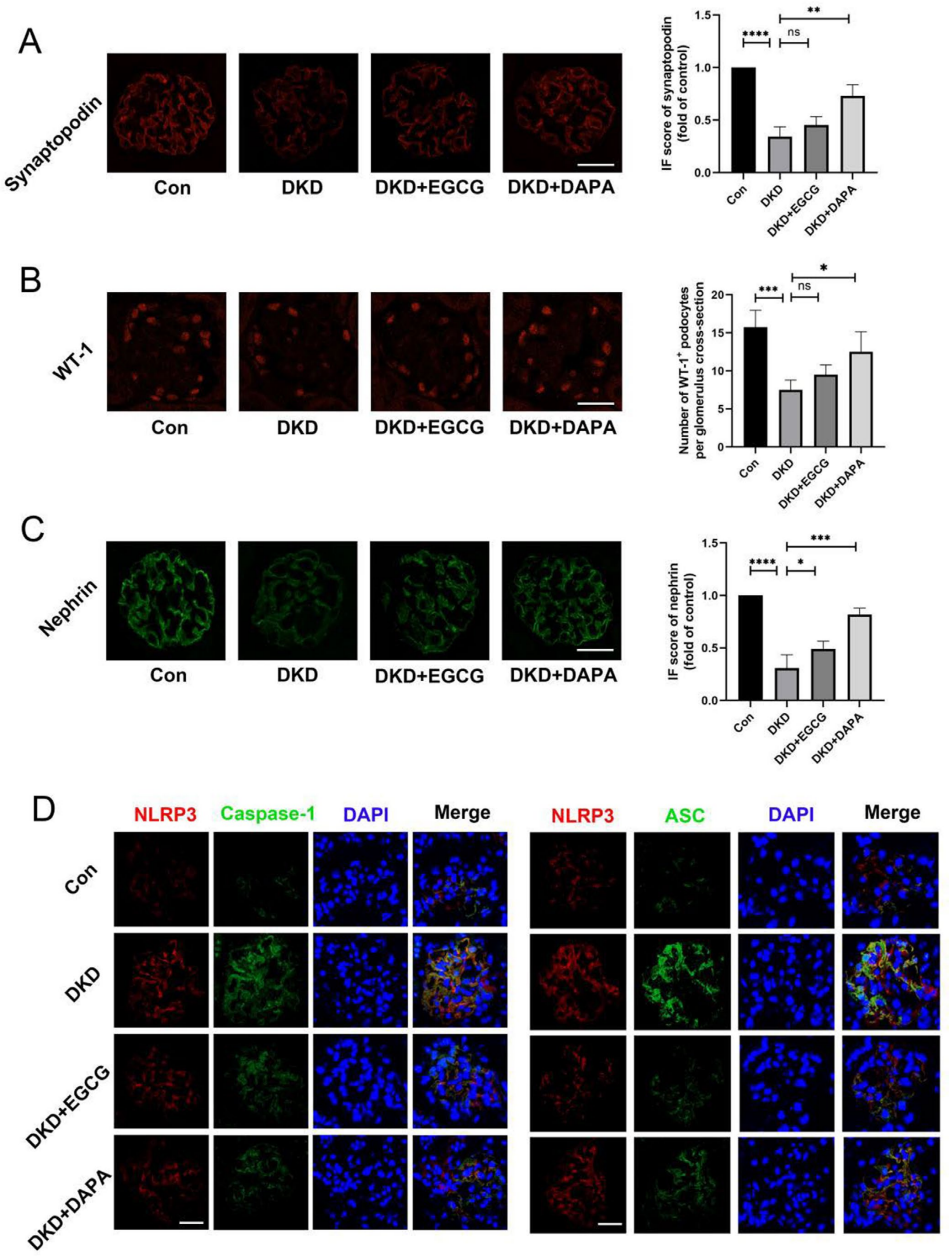
EGCG protects against glomerular and podocyte damage in the DKD mouse model. (A) Illustrative immunofluorescence staining and corresponding data demonstrating synaptopodin expression in kidney tissues across diverse groups (*n* = 4, bar = 20 μm). (B) Illustrative immunofluorescence staining and corresponding data illustrating WT‐1^+^ podocytes in kidney tissues among various groups (*n* = 4, bar = 20 μm). (C) Illustrative immunofluorescence staining and corresponding data depicting nephrin expression in the kidneys across various groups (*n* = 4, bar = 20 μm). (D) Illustrative immunofluorescence staining showing the co‐localization of NLRP3 with caspase1 and ASC in the kidneys among various groups (*n* = 4, bar = 20 μm). Values are shown as means ± SD, with significance levels indicated by ^****^ *p* < 0.0001, ***p < 0.001, **p < 0.01, *p < 0.05, ns: Not significant.

Given our previous demonstration that EGCG suppresses inflammation by modulating the TXNIP/NLRP3/IL‐1β axis in vitro, we hypothesized that EGCG might similarly reduce inflammation in DKD mice through this pathway. As displayed in Figure 6A–D, EGCG treatment decreased the levels of inflammatory factors TNF‐α, IL‐6, IL‐1β, and IL‐18, as well as the gene and protein expression of TXNIP, ASC, NLRP3, caspase1, and IL‐1β compared to untreated DKD mice, which aligned with our in vitro findings. Additionally, confocal microscopy revealed the co‐localization of NLRP3 with caspase1 and NLRP3 with ASC in vivo. The inflammasome components, including NLRP3, caspase1, and ASC, were remarkably elevated in the glomeruli of DKD mice compared with other groups. Furthermore, immunofluorescence analysis indicated a significant decline in the expression of glomerular synaptopodin, nephrin, and Wilms tumor 1 (WT1, a biomarker of podocytes) in DKD mice. EGCG treatment upregulated nephrin expression but did not significantly alter the levels of synaptopodin or WT1 (Figure 7A–D).

## 4 Discussion

Podocytes, as highly differentiated epithelial cells, are essential for the formation and preservation of the glomerular filtration barrier (Patrakka and Tryggvason 2009; Brinkkoetter, Ising, and Benzing 2013; Tian et al. 2014). Accumulating evidence has demonstrated that podocyte injury contributes to proteinuria and subsequent the development of DKD (Kalluri 2006; Ziyadeh and Wolf 2008; Mathieson 2011). However, the exact mechanism underlying podocyte injury remains unclear. Recent research, including our own studies, has emphasized the importance of sterile inflammation in podocytes during the progression of DKD. Moreover, the NLRP3 inflammasome is closely involved in sterile inflammation, which can be triggered by DKD‐related reactive metabolites and metabolic stimuli, including hyperglycemia, AGEs, and ROS. NLRP3 recruits and assembles with caspase‐1 and ASC, resulting in the activation of caspase1, which in turn cleaves IL‐1β and IL‐18 into their bioactive forms, triggering an inflammatory response and subsequent podocyte injury (Qiu and Tang 2016; Gu et al. 2019; Shahzad et al. 2022; Wu et al. 2021).

EGCG, the active compound in green tea, possesses diverse biological functions, including anti‐inflammatory and antioxidant properties (Kim, Quon, and Kim 2014; Lambert and Elias 2010). Man et al. found that EGCG ameliorates oxidative stress and inflammatory response in lipopolysaccharide‐stimulated endometritis via the SIRT1/NLRP3 pathway (Di et al. 2022). Similarly, Abigail et al. demonstrated that EGCG mediated the PI3K/Akt/mTOR pathway and alleviated the inflammation response in MRL/lpr mouse mesangial cells (Peairs et al. 2010). In this research, we first detected the protective effects of EGCG in vitro. We found that EGCG‐mediated protective effects against HG or AGEs induced the decrease of cell viability and the increase of apoptosis in podocytes. In addition, EGCG improved podocyte cytoskeleton rearrangement under HG. In vivo, EGCG protected kidney function, decreased albuminuria and body weight, and alleviated renal pathological injury. Then we detected the oxidative stress both in podoccytes and in DKD mice. We found that EGCG could decrease the ROS level and increase the mitochondrial membrane potential in podocytes under HG, increase SOD, CAT, and GSH, and decrease MDA levels in the DKD kidneys. Our results indicated that EGCG had protective effects on podocytes and DKD, which were consistent with other studies.

Reports have indicated that TXNIP, an endogenous inhibitor of TRX, is involved in the development of DKD (Advani et al. 2009; Shah et al. 2015; Song et al. 2020). Shi and co‐workers demonstrated that knockdown of TXNIP increased TRX expression, reduced ROS production, and decreased cell apoptosis and epithelial‐to‐mesenchymal transition induced by high glucose in mesangial and tubular epithelial cells (Shi et al. 2011; Wei et al. 2013). As a key binding partner of NLRP3, TXNIP is critical for the induction of the NLRP3 inflammatory complex. Studies have shown that inhibiting TXNIP can disrupt the TXNIP‐NLRP3 interaction, thereby preventing the subsequent stimulation of the NLRP3 inflammatory complex in podocytes (Dai et al. 2021; Abais et al. 2014). Furthermore, TXNIP is recognized as a crucial signaling molecule that connects oxidative stress to NLRP3 inflammasome activation in a ROS‐sensitive manner (Abderrazak et al. 2015).

Given that we have demonstrated EGCG's ability to suppress oxidative stress in podocytes, and with substantial evidence highlighting the importance of TXNIP in maintaining cellular redox homeostasis, we hypothesize that EGCG could attenuate oxidative stress and inflammation in podocytes and DKD through the TXNIP/NLRP3/IL‐1β axis. First, we evaluated the interactions between TXNIP and NLRP3 in podocytes and found that these interactions were obviously improved in podocytes under HG conditions. However, EGCG administration markedly blocked this enhanced TXNIP‐NLRP3 binding. We then assessed the TXNIP/NLRP3/IL‐1β axis both in vivo and in vitro. As expected, inflammatory factors such as TNF‐α, IL‐6, IL‐1β, and IL‐18, as well as signaling proteins including TXNIP, NLRP3, caspase1, ASC, and IL‐1β, were all upregulated.

Nevertheless, our research has few limitations. Firstly, the novelty of this research is limited, as the regulation of the NLRP3 inflammasome pathway by EGCG has been extensively studied. Secondly, the study lacks depth, as we did not explore how EGCG interacts with NLRP3 or TXNIP at a mechanistic level. In future research, we plan to conduct a more comprehensive investigation into the effects of EGCG on NLRP3.

**FIGURE 8:**
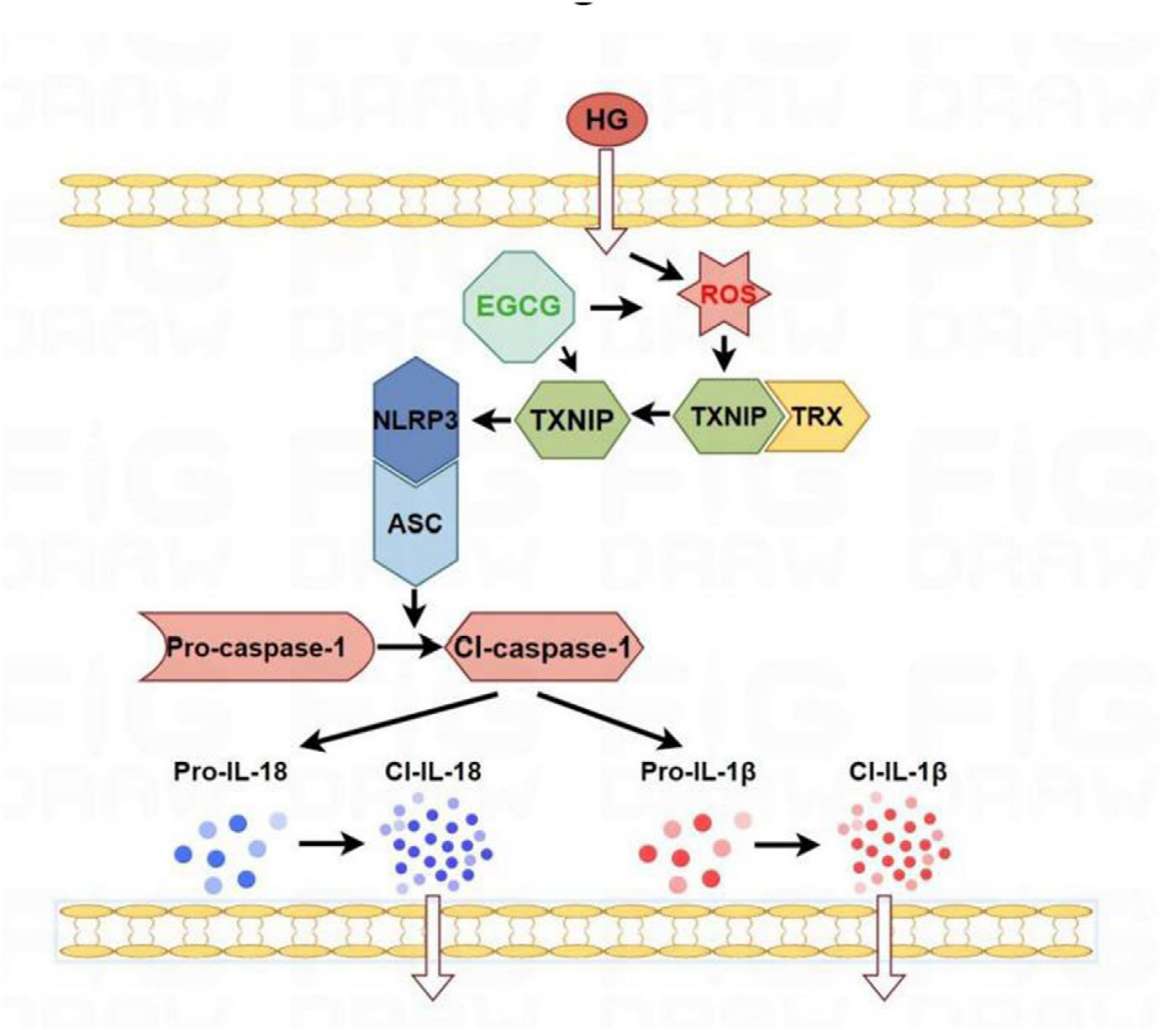
Visual summary. EGCG attenuates oxidative stress and inflammatory response in podocytes by suppressing the TXNIP/NLRP3/IL‐1β axis.

In summary, our findings demonstrated that EGCG could ameliorate oxidative stress and inflammation in podocytes and DKD through the TXNIP/NLRP3/IL‐1β axis. Our data indicate that EGCG protects against DKD by reducing inflammation via this pathway, suggesting that EGCG or green tea could be beneficial in managing DKD (Figure 8).

## Author Contributions

**Yinghui Wang:** conceptualization (equal), data curation (lead), formal analysis (lead), investigation (equal), methodology (lead), software (lead), validation (lead), visualization (lead), writing – original draft (lead), writing – review and editing (lead). **Qimeng Wang:** formal analysis (equal), methodology (equal), software (equal). **Mingming Wang:** methodology (equal). **Xueling Wang:** methodology (equal). **Qingzhen Liu:** software (equal). **Shasha Lv:** conceptualization (equal). **Huibin Nie:** software (equal). **Gang Liu:** funding acquisition (lead), project administration (lead), resources (lead), supervision (lead), writing – review and editing (supporting).

## Conflicts of Interest

The authors declare no conflicts of interest.

## Data Availability

Data supporting this study's findings can be obtained from the corresponding author upon request.
